# Myc Localizes to Histone Locus Bodies during Replication in Drosophila

**DOI:** 10.1371/journal.pone.0023928

**Published:** 2011-08-23

**Authors:** Kaveh Daneshvar, Abid Khan, Julie M. Goodliffe

**Affiliations:** Department of Biology, University of North Carolina at Charlotte, Charlotte, North Carolina, United States of America; Universita' di Milano, Italy

## Abstract

Myc is an important protein at the center of multiple pathways required for growth and proliferation in animals. The absence of Myc is lethal in flies and mice, and its over-production is a potent inducer of over-proliferation and cancer. Myc protein is localized to the nucleus where it executes its many functions, however the specific sub-nuclear localization of Myc has rarely been reported. The work we describe here began with an observation of unexpected, punctate spots of Myc protein in certain regions of Drosophila embryos. We investigated the identity of these puncta and demonstrate that Myc is co-localized with coilin, a marker for sub-nuclear organelles known as Cajal Bodies (CBs), in embryos, larvae and ovaries. Using antibodies specific for U7 snRNP component Lsm11, we show that the majority of Myc and coilin co-localization occurs in Histone Locus Bodies (HLBs), the sites of histone mRNA transcription and processing. Furthermore, Myc localizes to HLBs only during replication in mitotic and endocycling cells, suggesting that its role there relates to replication-dependent canonical histone gene transcription. These results provide evidence that sub-nuclear localization of Myc is cell-cycle dependent and potentially important for histone mRNA production and processing.

## Introduction

Myc protein controls metabolism, cell growth and proliferation in a coordinated fashion to provide energy production on demand and promote successful replication [1]. Myc functions by regulation of genes transcribed by RNA Polymerase II plus stimulation of transcription by RNA Polymerases I and III, helping to promote protein synthesis consistent with its primordial role in ribosome biosynthesis [2], [3], [4]. In keeping with its promotion of ribosome biogenesis, Myc influences nucleolar architecture via regulation of viriato (vito), the Drosophila Nol12 homolog that is required for nucleolar integrity during Myc stimulated growth [5].

We have previously shown variation in levels of Myc protein present during Drosophila embryogenesis [6], and during these experiments we observed punctate spots of Myc protein within the nuclei of embryonic cells. Interested in the potential significance of sub-nuclear Myc puncta, we investigated the identity of these and show here that Myc overlaps coilin and Lsm11 in the Histone Locus Body (HLB) of Drosophila.

The histone genes of *Drosophila melanogaster* exist as tandemly repeated sets of the canonical histone genes, which are transcribed during S phase of the cell cycle. The resulting replication-dependent histone transcripts lack a poly-A tail, rather the 3′ ends of histone mRNAs form a conserved stem-loop structure. Metazoans share this feature along with the U7 snRNP that binds the stem-loop, which includes proteins SLBP, Lsm10 and Lsm11 (reviewed in [7]). Lsm10 and Lsm11 are required for histone pre-mRNA processing and are found in the HLB, a nuclear body associated with the histone gene locus [8], [9]. Nascent histone transcripts associate with a Cyclin E/Cdk2 dependent phospho-epitope localized to the HLB [10]. We show that Myc associates with all HLBs that contain the same Cyclin E/Cdk2 phospho-epitope, and that Myc does not associate with HLBs in the absence of this epitope. Our results reveal a novel role for Myc as a cell-cycle dependent component of HLBs.

## Methods

### Genotypes

Oregon-R, except for embryos in Figures 3 and 4, which are doubly heterozygous for daughterless-Gal4 [11] and UAS-Lsm11-EYFP [9].

### Tissue fixation and immunostaining

Ovaries were fixed and stained according to Frydman and Spradling [12]; embryos were fixed and stained according to Sokac and Wieschaus [13]; larvae were fixed and stained according to Johnston and Edgar [14].

To be certain of the validity of the co-localization observed during this study, we obtained the following data from control experiments: single stainings for Myc showed similar puncta; stainings with each primary antibody combined with the wrong secondary showed no staining pattern except for one case, and we eliminated that secondary antibody. Staining with one primary antibody and all secondary antibodies together showed the same patterns as with one secondary alone. For microscopy we used sequential scanning of each channel, ensuring that detection of each fluorophore occurred only with the correct excitation laser.

### Antibodies

We used three different Myc antibodies (Santa Cruz, Figure S1). Immunostaining of larvae and embryos expressing an RNAi construct specific for dmyc [15] showed the absence of Myc puncta (Figure S2).

Primary antibodies were used at the following concentrations: rabbit anti-Myc 1∶500, goat anti-Myc 1∶250, mouse anti-fibrillarin 1∶1000 (abcam), guinea pig anti-coilin 1∶2000 (ovaries, embryos, the antibody was a gift from Joseph Gall) and 1∶500 (larvae), rabbit anti-Lsm11 1∶2000 (ovaries and embryos, gift from Joseph Gall) and 1∶500 (larvae), mouse anti-GFP 1∶500 (Covance), chicken anti-GFP (abcam) and mouse MPM-2 1∶1000 (Millipore).

### Microscopy

**Figure 1:**
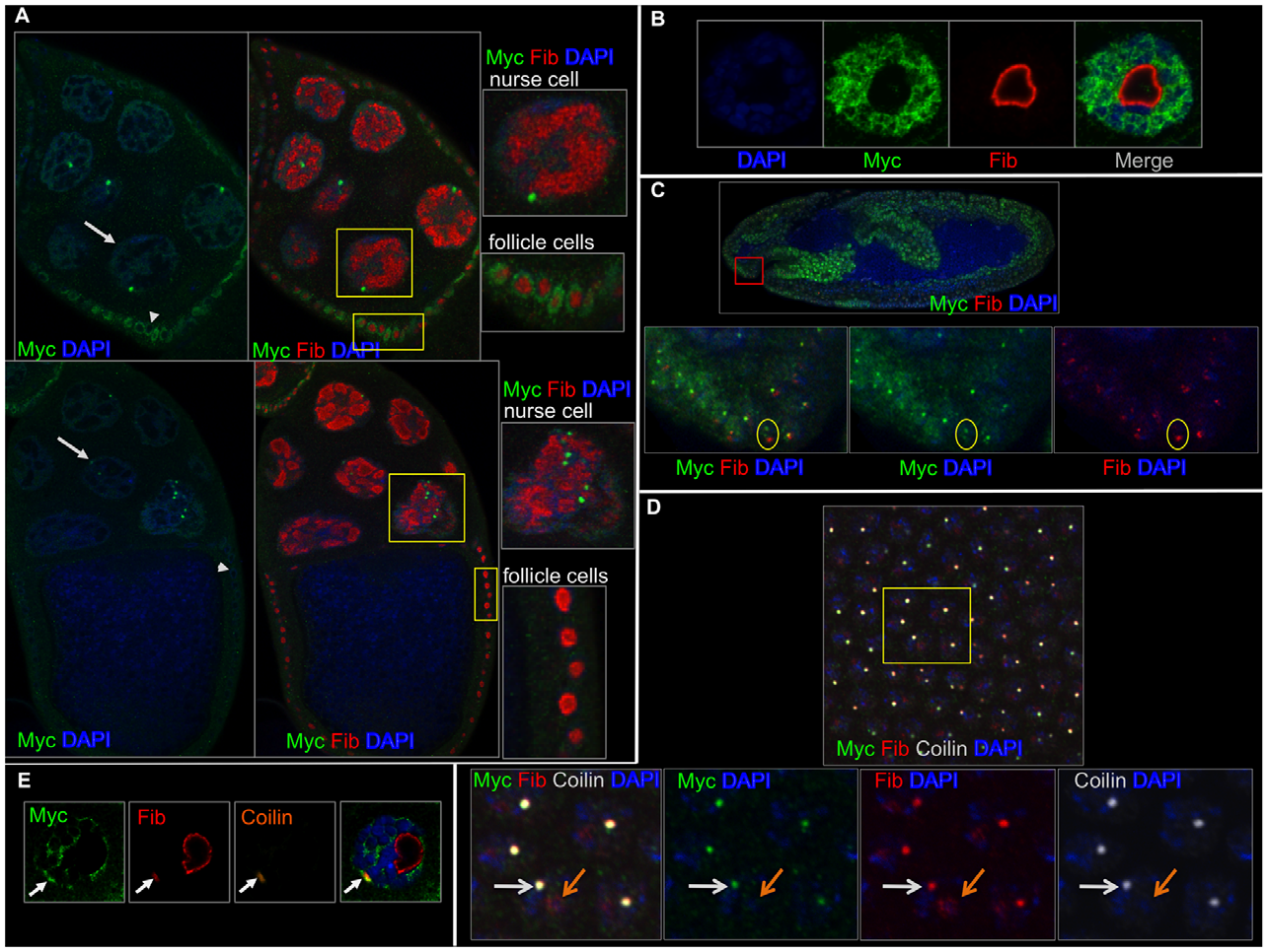
Myc does not localize to the nucleolus. A) Myc (green), fibrillarin (red) and DAPI label stage 8 (top) and 10 (lower) egg chambers. Arrows label a nurse cell, and arrowheads label a follicle cell. B) A larval salivary gland nucleus labeled with DAPI (blue), Myc (green) and fibrillarin (red) shows the exclusion of Myc from the nucleolus. C) A stage 10 embryo labeled with Myc (green), fibrillarin (red) and DAPI (blue) showing minimal overlap of Myc and fibrillarin (bottom three panels, note the cell within the yellow circles with a bright fibrillarin domain that lacks Myc). D) A stage 6 embryo labeled with Myc (green), fibrillarin (red), coilin (white) and DAPI, showing that locations where Myc and fibrillarin overlap are puncta containing coilin (shown by the white arrow in the higher magnification boxes below). Myc does not overlap with fibrillarin in the nucleolus (shown by the orange arrow). E) A larval salivary gland cell labeled with Myc (green), fibrillarin (red), coilin (orange) and DAPI, showing that Myc, fibrillarin and coilin overlap outside of the nucleolus (arrows).

Images were generated using a Zeiss LSM 710 or Olympus FluoView FV1000 confocal microscope. Images were acquired such that there were no saturated pixels, with minimal offset. Modifications to images were minor, and limited to gamma adjustment and contrast adjustments within the Olympus FV1000 software (Figure 1A was obtained on the Zeiss and we did not alter those images following acquisition). Modified images were cropped using Adobe Photoshop.

## Results

### Myc rarely localizes to the nucleolus

During the course of repeated antibody stainings to examine Myc protein levels, we observed clusters of cells containing punctate spots of Myc. As a transcription factor, the general nuclear localization that we observed in most cells was expected, however we were curious to determine the identity of the Myc puncta. Although *in vitro* experiments have not shown Myc to be associated with ribosomal DNA in Drosophila, Myc abundance correlates with the size and integrity of nucleoli [1], [16]. Therefore, we began our investigation by double staining ovaries, larval salivary glands and embryos with antibodies specific for Myc and fibrillarin, a marker for nucleoli.

In ovaries, we observed broad Myc accumulation with many Myc puncta located within the nuclei of nurse cells. Myc appeared excluded from nucleoli in both nurse cells and follicle cells. Different from the nurse cells, we observed no Myc puncta but general nuclear staining of Myc in follicle cells (Figure 1A). In the salivary glands of larvae, Myc localized to the nucleus and was largely excluded from the nucleolus (Figure 1B).

Fibrillarin is present in a sub-nuclear organelle known as the Cajal Body [9]. In embryos, Myc infrequently overlapped fibrillarin (Figure 1C, at stage 8, 63 Myc puncta also contained fibrillarin, n = 213). The overlap that we observed with Myc and fibrillarin in both larvae and embryos coincided with the Cajal Body, not the nucleolus (Figure 1D-E).

### Myc protein co-localizes with coilin

Cajal Bodies are organelles within the nucleus where the accumulation and some assembly of snRNPs occurs before mature snRNPs relocate to chromosomes for splicing [17]. They often appear adjacent to the nucleolus in Drosophila, and have been shown to transiently associate with several different loci in mammalian cells [9], [18]. The signature protein component of CBs is coilin; homozygous null coilin tissues lack CBs in Drosophila, and coilin knockout mice lack functional CBs [19], [20]. Given the presence of fibrillarin in CBs and the minimal overlap of Myc and fibrillarin, we next hypothesized that the Myc puncta were localized to CBs. Therefore, we double stained ovaries, larvae and embryos with anti-Myc and anti-coilin antibodies (a gift from Dr. Joseph Gall).

**Figure 2:**
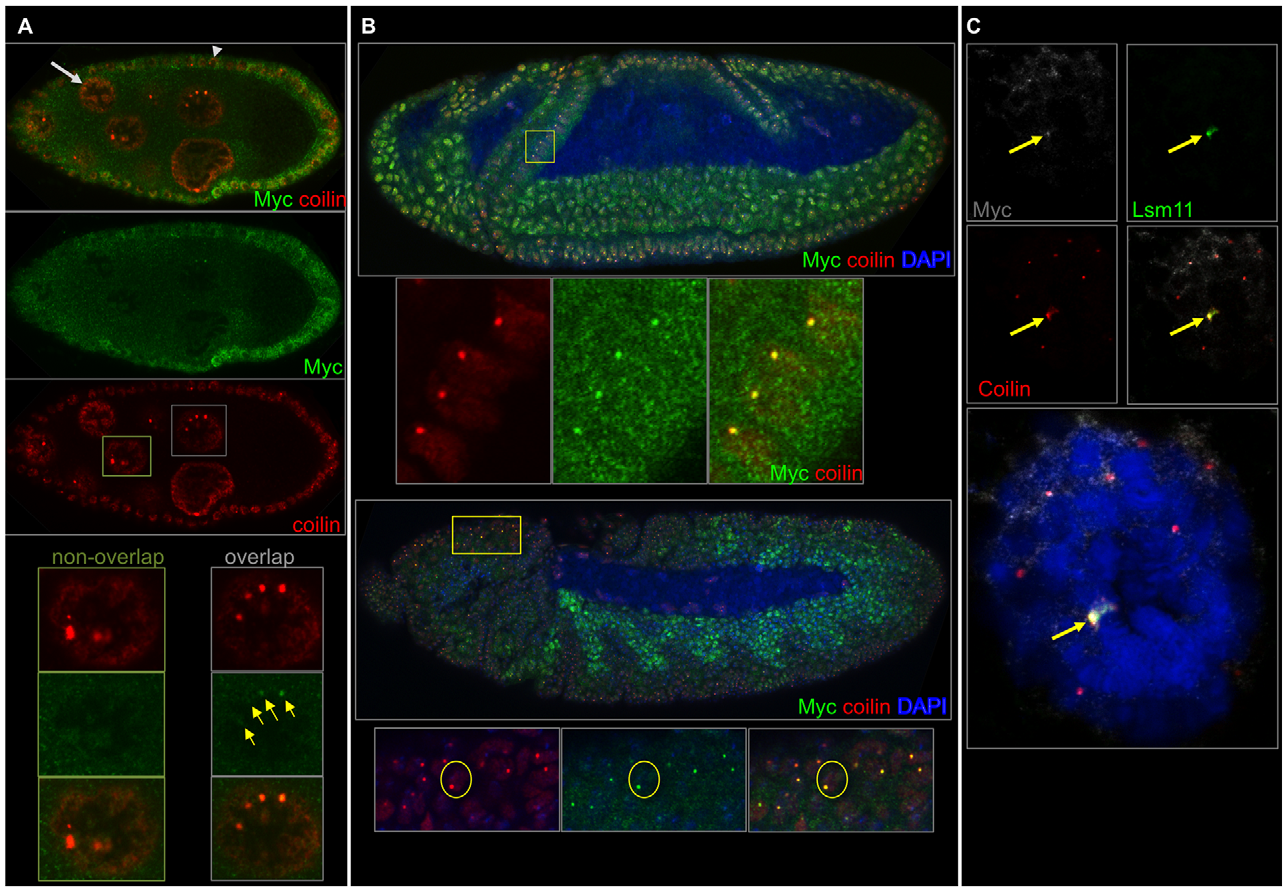
Myc and coilin overlap in ovaries, salivary glands and embryos. A) A stage 9 egg chamber labeled with Myc (green) and coilin (orange). The light gray arrow points to a nurse cell, and arrowhead points to a follicle cell. A nurse cell lacking overlap of Myc with coilin is shown (nurse cell in the green boxes, panels below and left), and a nurse cell with Myc and coilin containing puncta is also shown (nurse cell in the light gray boxes, panels below and right). B) A stage 8 embryo (top) and stage 11 embryo (bottom) labeled with Myc (green), coilin (red) and DAPI (blue). Myc is generally diffuse throughout nuclei at stage 8 except for parts of the cephalic furrow (higher magnification boxes below the top embryo; the region magnified is indicated in the yellow box) and future ectoderm. By stage 11, Myc appears in puncta of the ectoderm and head regions, the latter is shown magnified in the yellow box. These puncta contain coilin (see text). C) A larval salivary gland nucleus labeled with Myc (white), coilin (red), Lsm11 (green) and DAPI (blue) shows overlap of Myc with coilin in the largest coilin-containing body.

In ovaries, Myc and coilin localized to the same nuclear bodies in nurse cells. Beyond stage 2 of oogenesis, Myc bodies almost always contained coilin (49 in 50 Myc bodies contained coilin), although less than half of Cajal bodies contained Myc (21 Myc positive CBs, n = 57, Figure 2A). In the germarium and in follicle cells, Myc appeared diffuse and not obviously localized to any coilin-containing body. In salivary glands of third instar larvae, we observed co-localization of Myc with most of the large coilin bodies (Myc appeared in 22 out of 27 large coilin bodies, Figure 1E).

During embyrogenesis, Myc puncta appeared following the onset of cellularization. In the cellular blastoderm, Myc puncta almost always overlapped coilin (75 Myc positive CBs, n = 78 CBs). In cells of the early postblastoderm mitotic domains [21], Myc protein exhibited puncta that overlapped the CBs of those cells (39 Myc positive CBs, n = 41, Figure 2B, upper panels). Later, at approximately stage 11, Myc protein appeared diffuse in cells of the endoderm and the visceral musculature of the mesoderm. In the head regions and ectoderm, Myc protein appeared in puncta that overlapped CBs (32 Myc positive CBs, n = 37, Figure 2B, lower panels).

These data show that Myc and coilin co-localize broadly in Drosophila tissues, and the localization may correspond to CBs, HLBs or both. We investigated the possibility that the Myc and coilin overlap occurs in HLBs.

### Myc and coilin localization occurs mainly in HLBs

The HLB is a similar nuclear body to CBs, however it is distinct in that it contains the U7 snRNP and associates with the histone gene loci in Drosophila [9], [17]. HLBs and CBs can reside adjacent to each other or apart from each other, and both contain coilin [9], [19]. Because we observed Myc and coilin together in the nucleus, we investigated whether Myc puncta were HLBs rather than CBs. To visualize HLBs along with coilin, we obtained transgenic flies expressing an HLB marker, Lsm11-EYFP under the control of Gal4 (a gift from Dr. Gall). Lsm11 is a protein component of the U7 snRNP, which is specific for HLBs [17]. We induced expression of the transgene and triple-stained tissues with anti-Myc, anti-coilin and anti-GFP (embryos). In additional experiments, we stained wild type larvae and ovaries with anti-Lsm11 (also from Dr. Gall), anti-Myc and anti-coilin.

**Figure 3:**
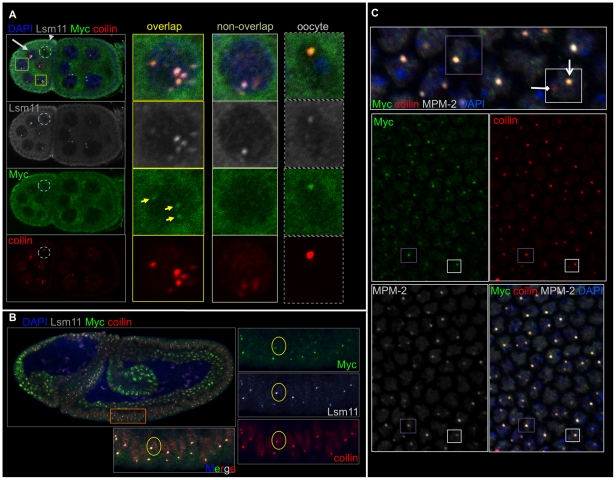
Myc localizes to Histone Locus Bodies (HLBs). A) Two egg chambers, ∼stages 5-6, labeled as indicated and showing that Myc, coilin and Lsm11 co-localize in nurse cells and the oocyte nucleus (the arrow labels a nurse cell and arrowhead labels a follicle cell; the oocyte nucleus is within the light blue dashed circle). The panels on the right show a nurse cell with Myc, coilin and Lsm11 in the same bodies; a nurse cell lacking Myc in a coilin-Lsm11 body; the oocyte with all three co-localized (right panels). B) A wild type, stage 8-9 embryo labeled with Myc (green), coilin (red), Lsm11-EYFP (white) and DAPI (blue) showing that Myc, coilin and Lsm11 co-localize to the majority of the bodies occurring in these embryos (lower panels show the cells in the orange box). C) The surface of an embryo at the onset of gastrulation, labeled with Myc (green), coilin (red) and MPM-2 (white) and DAPI, with a higher magnification of the merged image above. MPM-2 positive cells are replicating, and the MPM-2 bodies contain Myc and coilin (note cell in the purple boxes). One CB is evident with no MPM-2 or Myc present (in white box, HLB is labeled with an arrow, and the CB is labeled with a diamond-headed arrow).

**Figure 4:**
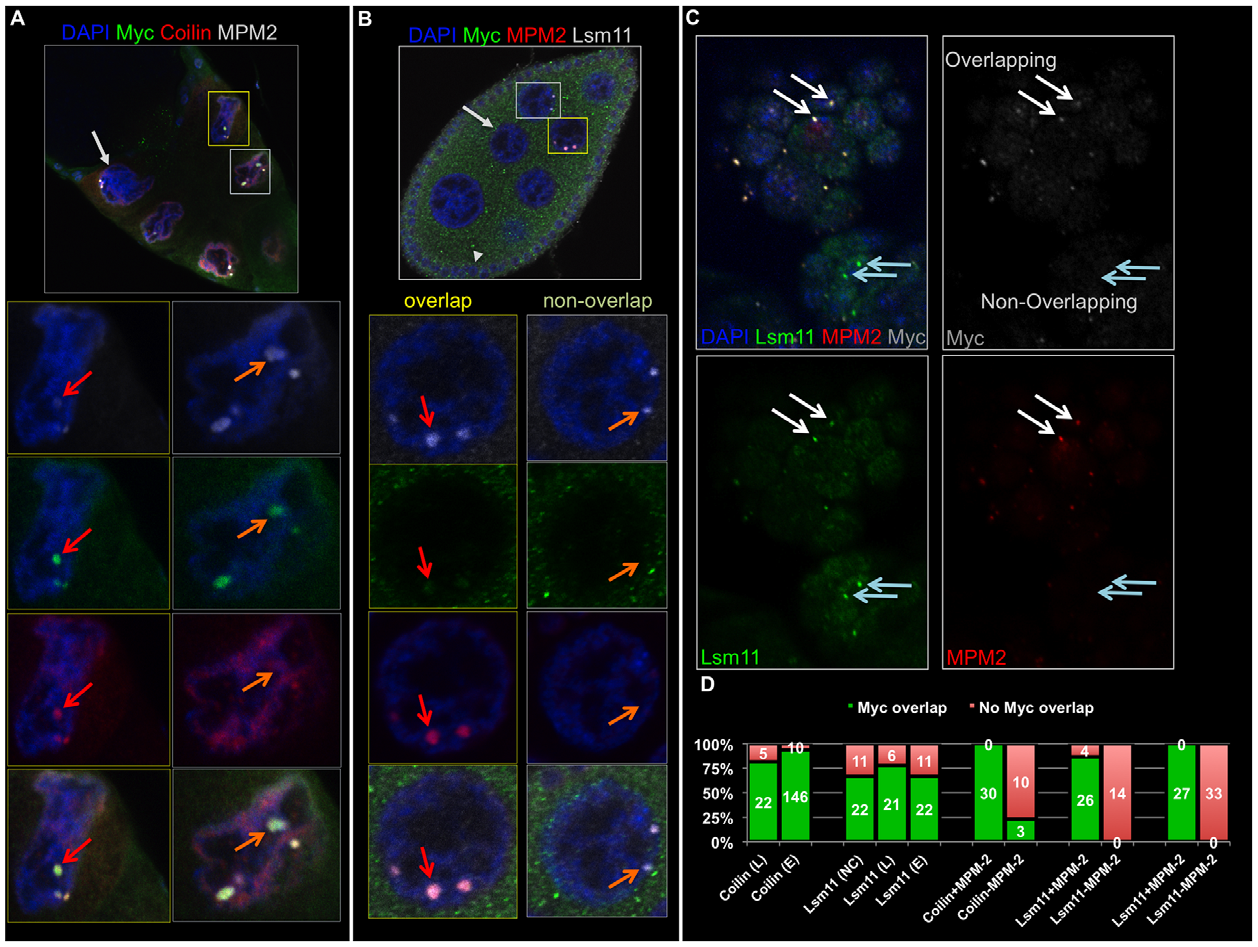
Myc localizes to HLBs in replicating cells. A) A stage 10 egg chamber is shown, labeled with Myc (green), coilin (red), MPM-2 (white) and DAPI, and a nurse cell is labeled with the light gray arrow. Myc, coilin and MPM-2 overlap in the HLB of the nurse cell in the yellow boxes, and MPM-2 and Myc overlap although coilin staining is weak in the HLB of the nurse cell in the gray boxes. B) A stage 8 egg chamber, labeled with Myc (green), MPM-2 (red), Lsm-11 (white) and DAPI shows that Myc puncta are the HLBs of replicating nurse cells. A nurse cell is shown with MPM-2 positive HLBs (cell in yellow boxes and magnified below), and Myc appears in those HLBs. A nurse cell is shown with Lsm11, non-MPM-2 staining HLBs, and Myc is absent (cell in light gray boxes, magnified below in the right-most panels). C) A germband-retracted embryo labeled with Myc (white), MPM-2 (red), Lsm11 (green) and DAPI showing that HLBs containing MPM-2 and Lsm11 also contain Myc (white arrows), and HLBs that contain Lsm11 but not MPM-2 do not contain Myc (light blue arrows). D) A chart showing the numbers reported in the text of Myc-overlapping (green bars) and non-Myc-overlapping puncta containing coilin (C), Lsm11 (L), and MPM-2 in embryos (E and the third and fifth sets of bars), larvae (L) and nurse cells (NC and also the fourth set of bars).

In Drosophila egg chambers, Myc overlapped coilin and Lsm11 in nurse cell nuclei (Figs. 3A and 4D). In salivary glands of third instar larvae, immuno-staining against Myc and GFP or Lsm11 showed the localization of Myc to HLBs; 81% of Lsm11 and coilin-containing HLBs included Myc (n = 27, Figures 2C and 4D). Myc localization to CBs containing coilin but no Lsm11 was less common; Myc appeared in 20% of non-Lsm11 Cajal Bodies, n = 29 (Figure 4D).

Similarly, in embryos, Myc puncta frequently contained both coilin and Lsm11 (Figure 3B), suggesting that the majority of embryonic Myc bodies are HLBs (in 221 Myc puncta, 208 also contained both coilin and Lsm11). The degree of Myc and Lsm11 overlap depended on the germ layer and/or region of the embryo. For instance, 86% of HLBs were Myc positive in the ventral ectoderm of stage 10 embryos (n = 117), however 13% of HLBs were Myc positive in the endoderm of the posterior midgut at the same stage (n = 52).

These data show that Myc occurs in the HLB of both mitotic and endoreplicating cells. However, Myc does not localize to HLBs in all nurse cells of an egg chamber, nor does it localize to all HLBs in the cells of an embryo.

### Myc localizes to HLBs only during replication

Because many different cell types showed Myc in the HLB, however not uniformly within an egg chamber or embryo, we investigated whether Myc localization to HLBs is cell cycle dependent. We stained embryos, larvae and ovaries with the monoclonal antibody MPM-2, which cross-reacts with phospho-epitopes of mitotic cells in many organisms [22]. In Drosophila embryos, MPM-2 recognizes the phospho-epitope of a protein present in HLBs, but only in cells with active Cyclin E/Cdk2 [10].

We first examined all coilin-containing bodies, which may be CBs or HLBs, by staining embryos with MPM-2, anti-coilin and anti-Myc antibodies. Myc appeared in 100% of the coilin and MPM-2 positive bodies, n = 30. Myc appeared in just 10% of CBs or HLBs lacking MPM-2, n = 30 (Figures 3C, 4D). We found similar results in ovaries, that Myc overlapped MPM-2 in all nurse cells containing puncta positive for MPM-2 (n = 30 nurse cells), and overlap with coilin was limited to MPM-2 positive bodies (all of which are HLBs later in oogenesis [19], as in Figure 4A).

To examine bodies identifiable as HLBs in cells undergoing replication, we stained ovaries and embryos with MPM-2, anti-Lsm11 (or anti-GFP) and anti-Myc. Myc and Lsm11 co-localized only in the presence of the MPM-2 epitope. HLBs containing both Lsm11 and MPM-2 were positive for Myc (Figure 4D). HLBs lacking the MPM-2 epitope also lacked Myc (Figure 4B and 4D). In embryos, replicating cells were identified with MPM-2 positive HLBs, and those bodies always included Myc (n = 27). Myc was never observed in MPM-2 negative HLBs, n = 33 (Figure 4C–D).

## Discussion

Our data show that Myc is a component of the HLB along with Lsm11 and the MPM-2 epitope-containing protein during replication, the time at which the canonical histone genes are transcribed. We observed little cell-type specificity of Myc puncta, since we identified Myc puncta throughout the embryo and in larval and ovary tissues. One exception is the lack of Myc puncta in the germarium and follicle cells of the ovary.

What could be the function or consequence of Myc in the HLB? One obvious possibility is that Myc helps boost transcription of the histone genes, consistent with its localization during replication when those genes are transcribed. In human embryonic stem cells and fibroblasts, HLBs contain the U7 snRNP in addition to a histone gene coactivator protein, p220^NPAT^, during mid-late G1 through S phase of the cell cycle [23]. These data suggest that HLBs are capable of histone gene transcription initiation. It is therefore logical to consider that Myc's role in the HLB is related to transcriptional activation. If this is indeed the case, Myc loss-of-function mutants should have decreased histone gene expression. Short-term knock-down of Myc by RNAi should address this question, as long as pleiotropic effects are minimized. The reciprocal should also be informative; over-expression of Myc may induce higher levels of histone gene expression. In our previous experiments expressing ectopic Myc [24], [25], we have not found dramatic changes in the levels of histone gene transcripts, however. Over-expression of Myc may not lead to increased levels of Myc in HLBs; that would have to be determined before conclusions can be drawn about the effect of elevated Myc on histone gene expression.

In human primary cells as in Drosophila, the U7 snRNP localizes to the HLB. In most human cancer cell lines, however, the U7 snRNP often localizes to the Cajal Body rather than the HLB, and therefore an intriguing delocalization of the U7 snRNP occurs in cancerous cells [18]. Elevated telomerase activity is a hallmark of cancer cells [26], and Cajal Bodies play a role in telomere length regulation; human telomerase RNA and the telomerase reverse transcriptase, hTERT, localize to CBs near telomeres during S phase [18]. Myc protein has been found to bind directly to TRF/PIN2, a DNA binding protein involved in telomere capping and telomerase inhibition. Expression of the TRF/PIN2 interaction domain of Myc, the protein's C-terminus that lacks its trans-activation domain, led to increased telomere length *in vivo* [27]. It would be informative to determine the localization of Myc with respect to HLBs, CBs and TRF/PIN2 in wild type and cancerous cells.

How might Myc be targeted to the HLB? Myc has been shown to be phosphorylated by cyclin E/Cdk2, altering Myc function, at mammalian c-Myc residue Ser-62 [28]. Drosophila Myc is not identical in this region of the protein, Myc Box I, however it does harbor a serine residue at the site next to the Ser-62 orthologous site [29]. An intriguing hypothesis is that cyclin E/Cdk2 phosphorylation of Myc causes subsequent localization to the HLB. Mutations eliminating potential phosphorylation sites of Myc in Drosophila would be informative in addressing this hypothesis. If a Ser-62 to alanine-62 mutant protein is unable to localize to the HLB, then phosphorylation of that site may have a role in Myc's localization to the HLB. Alternatively, ectopic cyclin E expression may drive constitutive localization of Myc in the HLB. We are pursuing this experiment.

Recently, White and colleagues identified several novel components of HLBs in Drosophila. Using biochemical and genetic approaches in S2 culture cells, the group identified Spt6 and the Drosophila NPAT homolog, Mxc, as novel components of the HLB. Myc was not identified as a component of the HLB in these experiments, but neither were two known components of HLBs: Lsm10 and Lsm11 [30]. Despite this fact, the new knowledge of HLB components, including Myc as described in this study, will help determine the function of HLBs, whose complete set of functions remains unclear.

## Supporting Information

Figure S1 MycN, dMyc and Myc-1 antibodies recognize Myc protein in embryos (top panels), egg chambers (middle panels) and larval salivary glands (bottom panels).
(TIFF)

Figure S2 The puncta recognized by anti-Myc are eliminated by RNAi specific for dmyc. We crossed hsGal4 females with UAS dmyc RNAi males, heat shocked third instar larvae (left panels) and embryos (right panels) for 90 minutes followed by fixation and staining (MycN antibody). Each column is a series of confocal sections through the same sample.
(TIFF)
