# Supplementary figures and images for: Myc Localizes to Histone Locus Bodies during Replication in Drosophila
Source: PLoS One. 2011 Aug 23;6(8):e23928. doi: 10.1371/journal.pone.0023928 (PMC3160328; doi:10.1371/journal.pone.0023928)

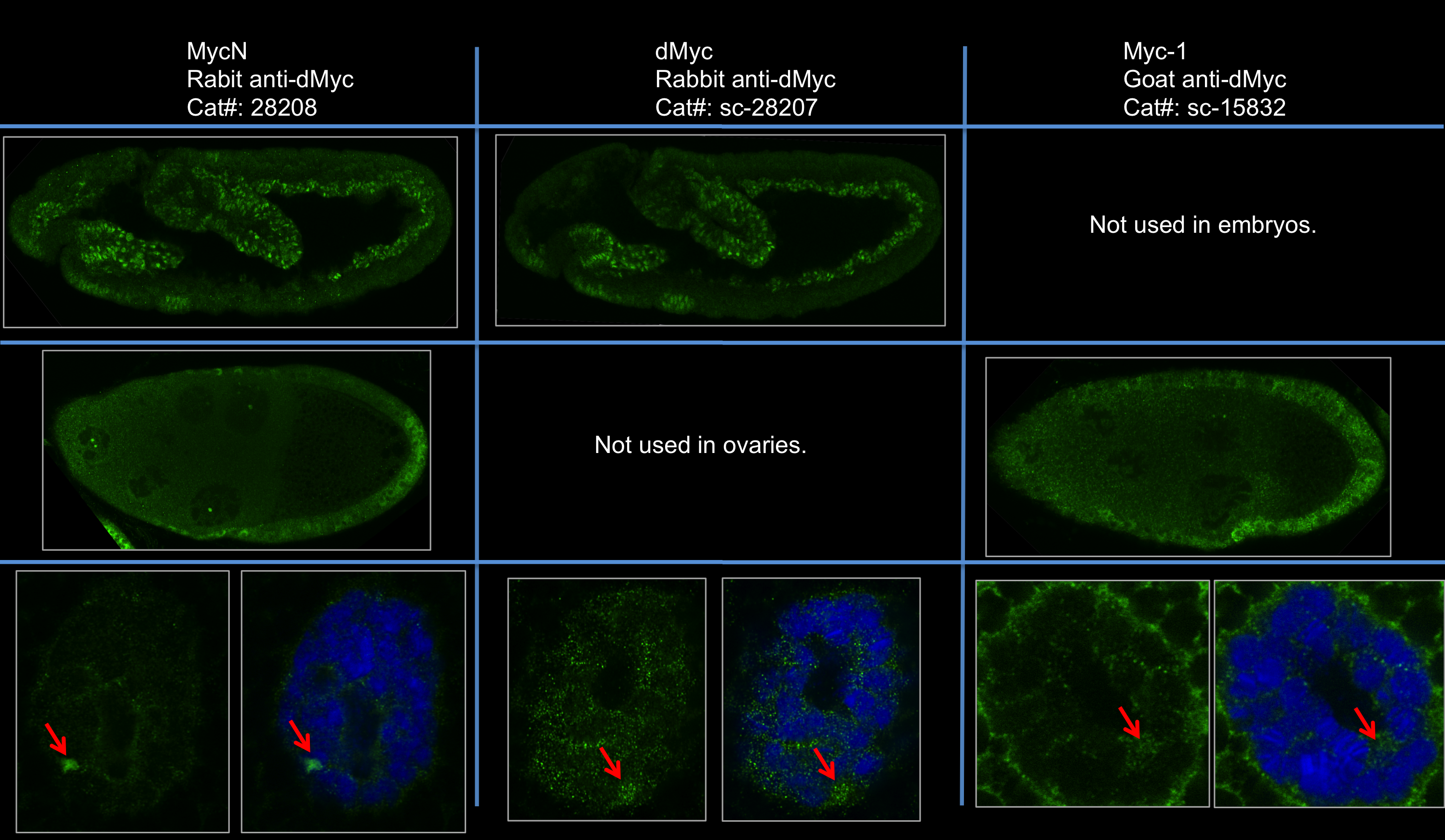

Supplement: Figure S1 — MycN, dMyc and Myc-1 antibodies recognize Myc protein in embryos (top panels), egg chambers (middle panels) and larval salivary glands (bottom panels). (TIFF) [file pone.0023928.s001.tiff]

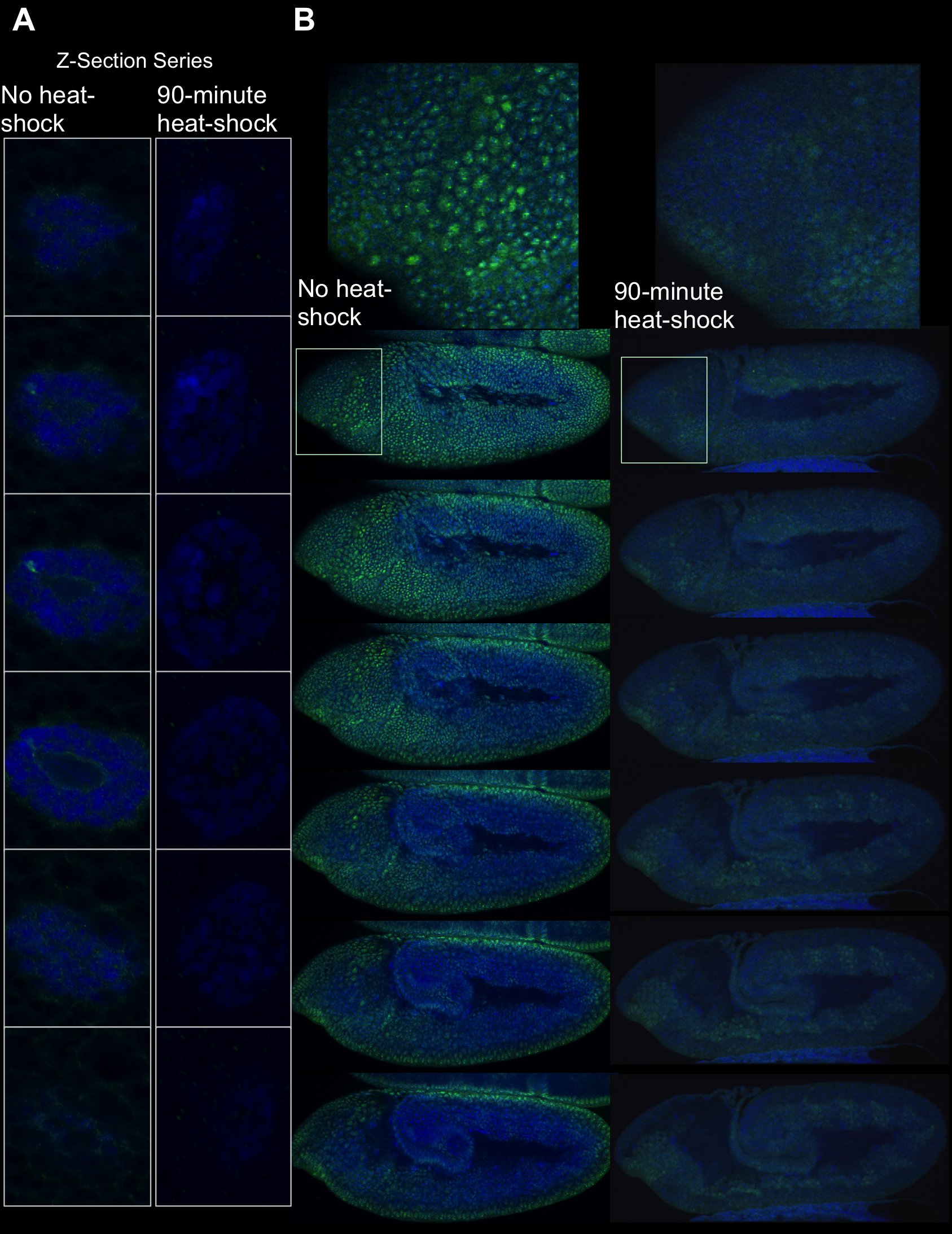

Supplement: Figure S2 — The puncta recognized by anti-Myc are eliminated by RNAi specific for dmyc. We crossed hsGal4 females with UAS dmyc RNAi males, heat shocked third instar larvae (left panels) and embryos (right panels) for 90 minutes followed by fixation and staining (MycN antibody). Each column is a series of confocal sections through the same sample. (TIFF) [file pone.0023928.s002.tiff]
